# Neuromuscular junction involvement in inherited motor neuropathies: genetic heterogeneity and effect of oral salbutamol treatment

**DOI:** 10.1007/s00415-023-11643-z

**Published:** 2023-03-04

**Authors:** Grace McMacken, Roger G. Whittaker, Ruth Wake, Hanns Lochmuller, Rita Horvath

**Affiliations:** 1 Department of Neurology, Royal Victoria Hospital, Belfast Health and Social Care Trust, Belfast, UK; 2 Translational and Clinical Research Institute, Newcastle University, Newcastle Upon Tyne, UK; 3 John Walton Muscular Dystrophy Research Centre, Institute of Genetic Medicine, Newcastle University, Newcastle Upon Tyne, UK; 4 Division of Neurology, Department of Medicine, Children’s Hospital of Eastern Ontario Research Institute, The Ottawa Hospital and Brain and Mind Research Institute, University of Ottawa, Ottawa, Canada; 5 Department of Clinical Neurosciences, John Van Geest Centre for Brain Repair, University of Cambridge School of Clinical Medicine, Level 3 A Block, Box 165, Cambridge Biomedical Campus, Cambridge, CB2 0QQ UK

**Keywords:** Inherited peripheral neuropathy, Charcot-Marie-Tooth disease (CMT), Distal hereditary motor neuropathy (dHMN), Genetic defects, Neuromuscular junction (NMJ), Neuromuscular transmission (NMT)

## Abstract

**Objectives:**

Inherited defects of the neuromuscular junction (NMJ) comprise an increasingly diverse range of diseases. Several recently identified genes highlight the overlap between peripheral neuropathies and congenital myasthenic syndromes (CMS). The beta-2 adrenergic receptor agonist salbutamol has been shown to provide symptomatic benefit in CMS, while improving structural defects at the NMJ. Based on these findings, we identified cases of motor neuropathy with NMJ dysfunction and assessed the effect of salbutamol on motor function.

**Methods:**

Cases of motor neuropathy with significant NMJ dysfunction, were identified using repetitive nerve stimulation and single fibre electromyography. Oral salbutamol was administered for 12 months. Repeat neurophysiological and clinical assessments were undertaken at baseline, 6 months and 12 months.

**Results:**

Significant defects of neuromuscular transmission were identified in 15 patients harbouring a range of genetic defects, including mutations in *GARS1, DNM2, SYT2* and *DYNC1H*. No clear benefit on motor function was seen following the administration of 12 months of oral salbutamol; however, there was a significant improvement in patient reported fatigue. In addition, no clear effect on neurophysiological parameters was seen in patients treated with salbutamol. Side-effects due to off-target beta-adrenergic effects were significant in the patient cohort.

**Conclusion:**

These results highlight the involvement of the NMJ in several subtypes of motor neuropathies, including subtypes of neuropathy due to deficits in mitochondrial fusion-fission, synaptic vesicle transport, calcium channels and tRNA synthetases. Whether the NMJ dysfunction is simply due to muscle reinnervation or a pathology unrelated to denervation is unknown. The involvement of the NMJ may represent a novel therapeutic target in these conditions. However, treatment regimens will need to be more targeted for patients with primary inherited defects of neuromuscular transmission.

## Introduction

Inherited motor neuropathies are a diverse group of disorders caused by a range of pathomechanisms leading to impairment of function of motor nerves. The main clinical manifestations are length dependent muscle weakness and wasting, which is predominantly due to axonal loss [1–3]. Several recent discoveries highlight the importance of dysfunction of the presynaptic nerve terminal and muscle denervation in the pathology of motor neuropathies. One recently discovered gene is *SYT2*, which encodes Synaptotagmin 2, a synaptic vesicle protein which functions as the main calcium sensor for neuromuscular transmission [4]. We identified heterozygous mutations in *SYT2* in two large kinships with an autosomal-dominant motor neuropathy syndrome and evidence of a neuromuscular transmission defect on clinical examination and neurophysiological tests [5, 6]. Recessive mutations in *SYT2* have also been shown to cause a severe, early onset form of congenital myasthenic syndrome (CMS) [7, 8]. In addition, we and others showed that mutations in the presynaptic choline transporter CHT1, encoded by *SLC5A7*, causes a presynaptic CMS [9–12]. Mutations in this same gene have also been shown to cause a motor neuronopathy [13, 14]. These findings suggest that presynaptic dysfunction may be a more general mechanism for peripheral axonopathies, and highlight the overlap between motor neuropathy and presynaptic CMS. Importantly, cardinal clinical features of a primary NMJ defect, such as fatiguability, ptosis, ophthalmoplegia and bulbar weakness, are absent in these motor neuropathy subtypes. This may reflect differences in the functional impact of mutations on motor nerve terminals.

Importantly, symptomatic treatments for NMJ dysfunction are readily available. These include acetylcholinesterase inhibitors such as pyridostigmine, which augment the synaptic response to the neurotransmitter acetylcholine, and 3,4-diaminopyridine, which blocks presynaptic potassium channels increasing calcium entry into presynaptic nerve terminals. In addition, beta-adrenergic agonists such as salbutamol, have become standard treatment for patients with CMS, and have been shown to lead to sustained improvements on muscle strength and are well tolerated in this patient cohort. In contrast to pyridostigmine and 3,4-diaminopyridine which have short-term effects on neurotransmission, animal studies demonstrate that long-term treatment with salbutamol leads to structural alterations at the end-plate including an increase in the postsynaptic folds, and can improve the NMJ abnormalities caused by genetic defects in *DOK7*, *MUSK*, and *COLQ* [15–17]. Together, this evidence suggests that the NMJ could play an important role in the pathogenesis of some motor neuropathies, and that treatments targeting the NMJ could provide symptomatic benefit in these conditions.

Given these recent discoveries, we sought to identify patients with neurophysiological evidence of NMJ dysfunction from a cohort of patients with inherited motor predominant neuropathies, and to assess the safety, tolerability and clinical and neurophysiological effects of salbutamol treatment on these cases.

## Methods

Patients were identified from the inherited neuropathy clinic at Newcastle Hospitals NHS Foundation Trust, which is the catchment area of a total population of 2.99 million. Inclusion of patients was based on the presence of a motor neuropathy/neuronopathy with no or only subclinical sensory changes on electrophysiology. Acquired causes were excluded by detailed laboratory analysis and lack of response to immunosuppressive therapy.

At the time of neurophysiological assessment, patients were assessed for muscle weakness and fatigability. Participants were assessed using the CMT symptom score (CMTSS) and CMT examination score (CMTES), which are subscores of the CMT neuropathy score (CMTNS), taking into account sensory and motor symptoms and signs in upper and lower limbs [18]. ​​Maximal voluntary isometric contraction (MVIC) was also measured with a myometer (Cit Technics, Haren, The Netherlands), for distal arm (hand-grip, three-point pinch) and leg (foot dorsiflexion) strength. In addition, patients completed the Individualised Neuromuscular Quality of Life (INQoL) questionnaire.

At their baseline visit, participants were commenced on oral salbutamol at a dose of 12 mg daily in 3 divided doses, based on the typical dose used for patients with primary NMJ defects such as congenital myasthenic syndromes. Treatment was administered for 12 months. Safety and tolerability were assessed at each patient visit by physical examination and clinical history taking.

Repetitive nerve stimulation (RNS), single fibre EMG (SFEMG) and clinical assessments were repeated at 6 months and 12 months. RNS and SFEMG was repeated by the same neurophysiologist using the same muscle groups. RNS involves stimulation of motor nerve via surface electrodes and measurement of the resulting summated response, known as the compound muscle action potential (CMAP). In patients with defects of neuromuscular transmission (NMT), the compound muscle action potential (CMAP) amplitude can vary with repetitive stimulation. A reproducible percentage change or “decrement” of 10% between the first and fourth CMAP amplitude is highly indicative of an NMJ disorder. SFEMG is a more sensitive test and can detect milder defects of NMT, as it measures the variability (or “jitter”) in the time taken to excite the muscle fibre.

Participants provided written informed consent, approved by Newcastle and North Tyneside Local Research Ethics Committee for this study (16/NE/0161). Anonymized data not published within this article will be made available by request from any qualified investigator.

## Results

### Patient cohort

15 individuals, from 8 families, with motor neuropathy were included. Clinical characteristics are outlined in Table 1, with age listed at first assessment. Genetic diagnosis was confirmed in ten patients. Five remained genetically undiagnosed despite all having undergone whole exome sequencing. One patient (#5) was lost to follow-up, and another two (#1 and #14) withdrew from the study after the baseline visit due to inability to tolerate neurophysiological testing and pregnancy respectively. All other patients underwent three serial clinical and neurophysiological assessments at baseline, 6 and 12 months.

**Table 1:** Baseline characteristics of participants

Patient #	Gene	Mutation	Age (y)	Sex	Disease Duration (y)	Duration of treatment (w)
*1	*DYNC1H1*	Heterozygous *c.9233G* > *A, p.(Arg3078Gln)*	47	F	43	Withdrawn
2	*GARS1*	Heterozygous *c.1528A* > *C, p.(Lys510Gln)*	53	F	47	52
3	*DNM2*	Heterozygous *c.1789 T* > *A, p.(Tyr597Asn)*	52	F	35	52
4	Unknown		44	M	9	52
*5	*SYT2*	Heterozygous *c.923C* > *T, p.(Pro308Leu)*	25	M	15	Withdrawn
6	*SYT2*		29	F	24	52
7	*SYT2*		49	F	44	52
8	Unknown		51	F	30	52
9	Unknown		49	M	39	4
10	Unknown		47	M	35	52
11	Unknown		75	F	60	52
12	*DYNC1H1*	Heterozygous *c.1834G* > *A, p.(Val612Met)*	31	M	24	52
13	*GARS1*	Heterozygous *c.979G* > *A, p.(Gly327Arg)*	19	M	5	8
*14	*GARS1*	Heterozygous *c.1528A* > *C, p.(Lys510Gln)*	31	F	26	Withdrawn
15	*DNM2*	Heterozygous *c.1789 T* > *A, p.(Tyr597Asn)*	20	F	10	52

Patients marked * left study

*AA* amino acid, *w* weeks, *y* years

### Clinical features

All patients had features of length dependent motor neuropathy with distal weakness, atrophy, and areflexia. Patients 2, 13 and 14 who had dominant mutations in *GARS1*, and one genetically undiagnosed patient (#4) had an upper limb predominant pattern of weakness (onset of weakness and wasting in distal upper limbs prior to lower limb involvement) [19]. Patients were assessed for fatigable weakness and symptoms in-keeping with NMJ dysfunction. No patients had myasthenic-like symptoms such as symptom fluctuation or exacerbating factors, or signs of fatigability on examination.

### Neurophysiological features

NMJ function was assessed using RNS and SFEMG. RNS was found to be normal (≤ 10%) in all patients except patient 6 (*SYT2* mutation) who had a decrement of − 15% in APB at 3 Hz. The more sensitive test of NMJ dysfunction, SFEMG, demonstrated abnormal jitter (≥ 10% fibre pairs) in all patients. The most severe NMJ abnormalities were in patients with mutations in *SYT2*, *GARS1* and *DNM2* as well as two genetically undiagnosed cases.

### Response to oral salbutamol

All participants were commenced on 4 mg oral salbutamol three times a day at baseline visit. Two patients (#9 and #13) discontinued treatment after 4 and 8 weeks respectively, due to intolerable muscle cramps. Patients 1, 5 and 14 withdrew from the study after the baseline visit. The remaining 10 patients continued on 4 mg salbutamol three times daily for 12 months.

Overall, the CMTES, CMTSS and myometry assessments declined at a level which was comparative to natural history data from CMT patients (Table 2) [20, 21]. Two patients (both genetically undiagnosed) reported subjective improvement following salbutamol treatment. Patient 4 reported subjective improvement in upper limb function, and manual muscle testing (MMT) in distal upper limbs showed a change in dominant abductor pollicis brevis (APB) strength from MRC grade 2 at baseline and 6 months to MRC grade 4 at 12 months. Patient 8 reported improved energy levels and proximal upper and lower limb strength. There was no improvement in objective strength assessments in this case, however, the Individualised Neuromuscular Quality of Life (INQoL) index, the composite score from each of the 5 sections of the INQoL, was used to assess symptom specific impact on quality of life at each study visit, with a higher index indicating greater symptom impact. The mean INQoL showed an increase over the 12-month study, with scores particularly increasing in the categories of locking and pain. There was, however, a significant improvement in participant reported fatigue at 12 months compared to baseline (INQoL fatigue score 59.30 at baseline [95% confidence interval (CI) 53.12–65.48] and 37.19 after 12 months salbutamol [95% CI 29.44–44.94].

**Table 2:** Clinical outcome measures at baseline and following 6 and 12 months of daily salbutamol treatment

	Baseline (mean ± S.D, *n* = 15)	6 months (mean ± S.D, *n* = 10)	12 months (mean ± S.D, *n* = 10)
CMTES	7.80 ± 2.78	7.85 ± 2.94	8.08 ± 3.01
CMTSS	3.40 ± 1.55	3.69 ± 1.70	3.92 ± 1.71
MVIC (N)			
Hand grip	65.80 ± 24.10	63.60 ± 25.05	60.13 ± 26.50
Three-point pinch	60.33 ± 28.42	59.57 ± 28.52	58.07 ± 28.79
Foot dorsiflexion	53.74 ± 24.61	50.93 ± 20.82	47.13 ± 21.40
INQoL index			
weakness	56.14 ± 23.34	56.49 ± 26.50	57.89 ± 24.77
pain	49.12 ± 25.06	53.68 ± 28.77	**56.84 ± 27.65
fatigue	59.30 ± 11.16	*50.53 ± 21.95	***37.19 ± 13.98
locking	14.74 ± 10.37	***26.67 ± 10.61	***30.18 ± 8.78
activities	55.78 ± 18.10	55.11 ± 19.59	55.56 ± 19.26
dependence	43.70 ± 17.68	44.18 ± 16.06	45.19 ± 12.12
social relationships	40.67 ± 21.04	40.89 ± 19.83	41.22 ± 20.16
emotion	36.96 ± 16.19	38.15 ± 15.80	38.51 ± 18.27
body image	47.41 ± 23.56	48.15 ± 25.60	24.63 ± 28.16
Total	44.23 ± 9.77	45.01 ± 10.99	45.18 ± 11.44

INQoL scores for each domain are presented as a percentage, with higher scores indicating greater symptom impact

*CMTES* Charcot Marie Tooth examination score, *CMTSS* Charcot Marie Tooth symptom score, *INQoL* individualised quality of life neuromuscular questionnaire, *MVIC* maximum voluntary isometric contraction

*P < 0.05, **P < 0.01, ***P < 0.001, paired Student’s *t* tests

**Fig. 1:**
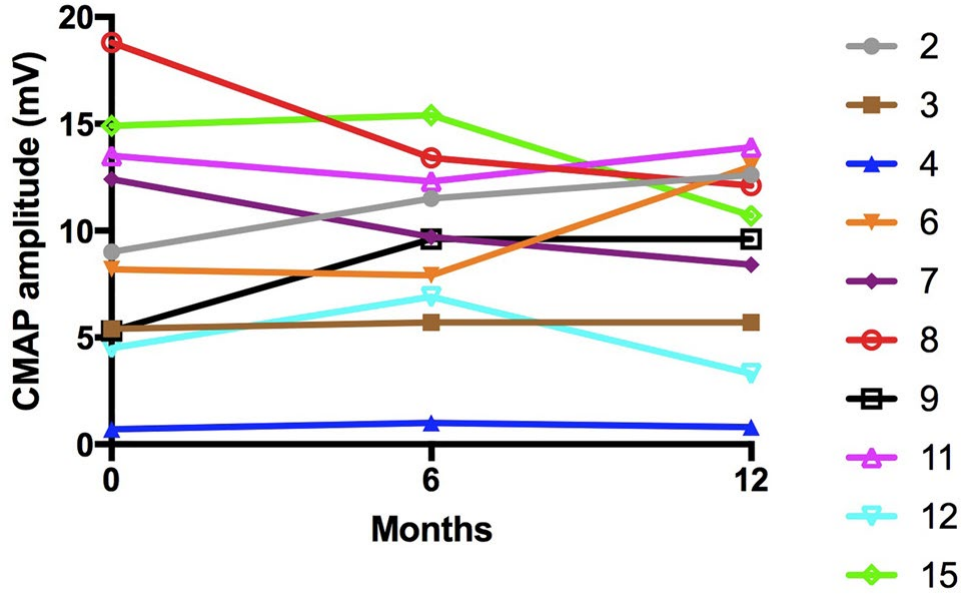
CMAP amplitude changes in abductor pollicis brevis (APB) in 10 patients treated with salbutamol

**Fig. 2:**
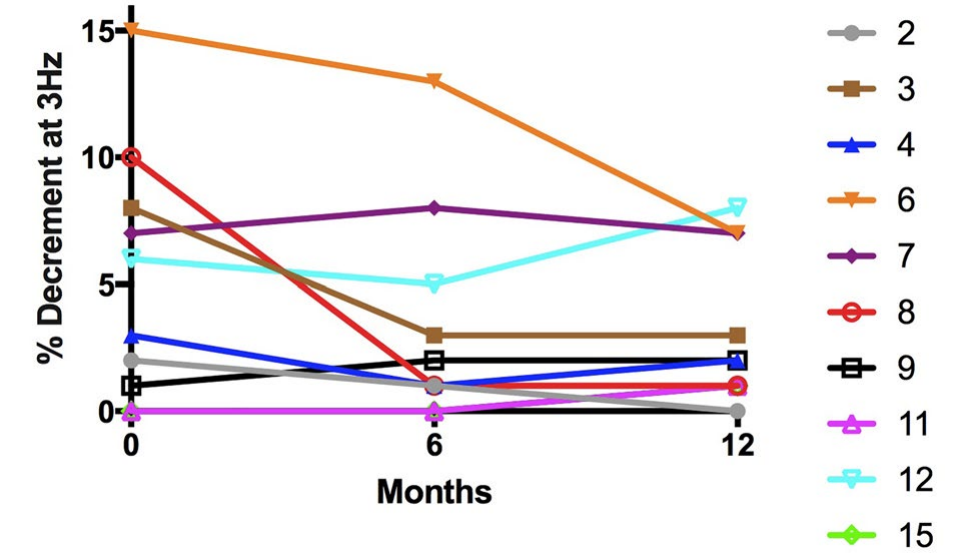
Change in decrement on repetitive nerve stimulation of median nerve (APB) in patients treated with salbutamol

**Fig. 3:**
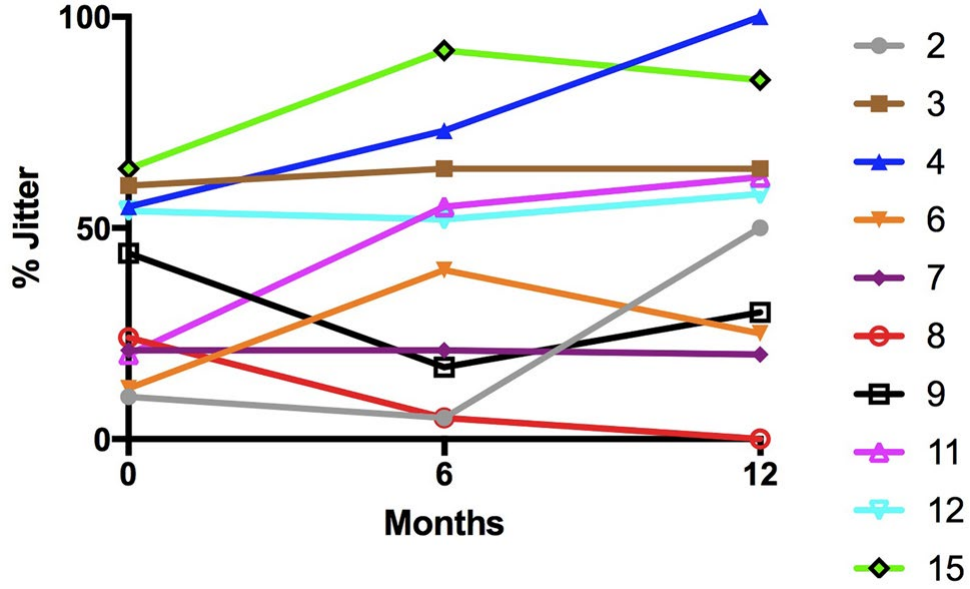
Change in % jittering fibre pairs in extensor digitorum communis (EDC) muscle in patients treated with salbutamol

The neurophysiological changes in the 10 patients who completed 12 months of salbutamol treatment are summarised below. Distal CMAP amplitudes of the abductor pollicis brevis (APB) were uniformly low and did not significantly change over the course of the study (Fig. 1). The change in NMJ abnormalities was assessed using RNS and SFEMG. The only patient with abnormal decrement of CMAP amplitude on RNS (patient 6) did show a reduction in this decrement during salbutamol treatment (13% decrement and 7% decrement after 6 months and 12 months of salbutamol treatment, respectively) (Fig. 2). However, comparison of the more sensitive test of SFEMG at baseline, 6 months and 12 months did not show any significant difference in % jitter or blocking in either extensor digitorum communis (EDC) or tibialis anterior muscle in patients treated with salbutamol (Fig. 3). There was no correlation between the degree of jitter or blocking and manual muscle strength MRC scores or myometry assessment.

All participants reported side effects from salbutamol treatment. The most common side effect was cramping of distal lower and upper limb muscles, particularly at night, and was reported in all patients. This side effect tended to be persistent throughout the duration of the study. Two participants discontinued treatment due to the severity of these leg cramps. Other reported side effects included insomnia, palpitations and tremor. No serious adverse events occurred.

## Discussion

Defects at the NMJ are increasingly demonstrated in various types of neuromuscular disorders, as well as myasthenic disorders, in which the primary site of the pathological defect is the NMJ. NMJ dysfunction has been demonstrated in amyotrophic lateral sclerosis, spinal muscular atrophy, inherited myopathies and muscular dystrophies, and in mitochondrial disease [22–25]. NMJ dysfunction is also implicated in disorders of motor nerves.

Here, we present a cohort of patients with inherited motor neuropathies with evidence of NMJ dysfunction on neurophysiological testing. Three cases had CMT2D, caused by dominant mutations in Glycyl tRNA synthetase (*GARS1*), a ubiquitously expressed enzyme which is essential for protein translation. Two cases had mutations in *DYNC1H1*, which encodes cytoplasmic dynein heavy chain 1, the primary protein responsible for retrograde axonal transport in neurons. Two cases presented with CMT2M due to dominant mutations in *DMN2*, encoding the ubiquitously expressed GTPase dynamin 2, which is involved in endocytosis and intracellular membrane trafficking [26]. In addition, the patient cohort included the previously described cases with heterozygous mutations in *SYT2*, encoding synaptotagmin 2, the main calcium sensor at the presynapse [4, 6]. Interestingly, NMJ defects have also been demonstrated in several animal models of these subtypes of motor neuropathies early in the course of the disease. The Gars^C201R^ mouse, exhibits smaller and fragmented end-plates which is independent of innervation status of the NMJs, and occurs prior to the loss of motor neuron connectivity [27–29]. The *Cramping1 (Cra1/* +*)* mouse has hypomorphic mutations in *DYNC1H1* [30]. *Cra1/* + mice exhibit morphological NMJ defects at the onset of muscle weakness, and prior to loss of motor or sensory neurons [31]. In addition, dominant mutations in *DMN2*, which can cause centronuclear myopathy or axonal CMT, have been shown to lead to severe alterations at the NMJ in animal models of both the myopathy and neuropathy phenotypes [32, 33].

The majority of disorders in which the NMJ is the primary site of pathology (e.g. autoimmune myasthenia gravis, CMS and Lambert-Eaton myasthenic syndrome) respond to symptomatic treatment with one or more drugs which may alter neuromuscular transmission, including pyridostigmine, salbutamol or 3,4-diaminopyridine (3,4 DAP). We previously showed that patients with *SYT2* mutations responded clinically to 3,4-DAP (which blocks potassium channels thereby increasing calcium entry into presynaptic nerve terminals); 3,4-DAP lead to symptomatic improvement, improvement in timed tests and also led to reduction in jitter on SFEMG [5]. In addition, treatment of the Gars^C201R^ mouse with acetylcholinesterase inhibitors resulted in improved functional tests and enhanced synaptic currents [29]. The detection of NMJ defect in SMA [34] and ALS [35] led to clinical trials with NMJ targeting drugs including Salbutamol with some positive effect [36]. We believe that our study is important, as it investigated the potential benefit of a safe and widely used drug in dHMN, where no effective treatments are available.

Here, we sought to assess the feasibility and tolerability of treatment of this cohort with oral salbutamol, a β2 agonist which is known to improve the structural and functional integrity of the NMJ. We did not identify any clear benefit from salbutamol treatment over this 12-month study in assessments of muscle strength or in neurophysiological tests. However, despite a lack of objective evidence, this patient cohort did report a significant and sustained improvement in fatigue following 6 months and 12 months of oral salbutamol. The mechanism for fatigue in CMT is not known, however the ergogenic effects of β2 agonists may lead to increased endurance through central or peripheral mechanisms [37, 38]. Fatigue has been previously shown to be a disabling symptom in CMT and to significantly impact on quality of life [39]. Therapeutic improvement in patient reported fatigue therefore has the potential to lead to substantial improvement in the lives of people with CMT. Interestingly, all patients reported an increase in painful cramps in distal muscles whilst taking salbutamol. We were unable to assess whether these cramps were myogenic or neurogenic in origin. In our experience, this side effect is rarely reported in patients with CMS taking salbutamol.

We accept that 12 months of follow-up may be too short a time to detect an impact of salbutamol treatment on the clinical and neurophysiological parameters we measured, as has been indicated by clinical trials in CMT [40]. In addition, the majority of patients had a prolonged disease duration of several decades at the time of commencing the study, which is likely associated with significant axonal loss. It may be that treatments to improve the function of NMJs in these patients would be more beneficial early in the disease course, the point at which NMJ dismantling is most implicated. Currently, several experimental treatments which alter the expression of NMJ proteins have been shown to have benefit in pre-clinical studies of neuromuscular disorders, including CMS, muscular dystrophy and motor neuron disease. Dok7 is a postsynaptic cytoplasmic protein which is essential for activation of muscle-specific receptor kinase (MuSK) and subsequent postsynaptic differentiation [41]. Administration of an adeno-associated virus (AAV) vector encoding *DOK7*, has been shown to increase muscle strength and extended survival in models of *DOK7* CMS, autosomal dominant Emery-Dreifuss muscular dystrophy, and amyotrophic lateral sclerosis due to mutations in *SOD1* [42, 43]. In addition, treatment of a mouse model of spinal muscular atrophy (SMA) with a soluble fragment of the synaptic organiser protein agrin, lead to improved motor performance and increased life span [44]. These results suggest that upregulation of proteins involved in NMJ development and stabilisation may have therapeutic benefit in many neuromuscular disorders, including those pathologically characterised by axonal loss and motor neuron cell death.

We accept that this study has several limitations. The sample size was small, and several patients were unable to complete the study. Given that inherited motor neuropathies are clinically heterogenous and exhibit variable rates of disease progression, recruitment of a larger cohort or a genetically homogenous cohort may be better able to detect any alteration in progression due to salbutamol. We also did not include a placebo group for comparison of those treated with salbutamol in this pilot study, due to the not neglectable side effects of the salbutamol. In addition, some participants remain genetically undiagnosed and it is possible that these cases could have acquired neuropathies or other neuromuscular conditions mimicking inherited neuropathies. However, our study indicates that NMJ dysfunction is common in inherited motor neuropathies. Further studies treating larger patient cohorts, potentially earlier in the disease process, will be required to ascertain the impact of therapeutic modulation of neuromuscular transmission on these conditions.

## Data Availability

The private information on patients presented here is stored in the Newcastle upon Tyne Hospitals NHS Trust. Genetic data are stored within the UK Genetic Testing Network`s database. Whole exome sequences can be found in the RD-CONNECT database.
